# Supplementary material for: Bacterial encapsulins as orthogonal compartments for mammalian cell engineering
Source: Nat Commun. 2018 May 18;9:1990. doi: 10.1038/s41467-018-04227-3 (PMC5959871; doi:10.1038/s41467-018-04227-3)
Supplement: Supplementary file 1 — Supplementary Information [file 41467_2018_4227_MOESM1_ESM.pdf]

Bacterial encapsulins as orthogonal compartments for mammalian cell engineering

Felix Sigmund, Christoph Massner, Philipp Erdmann, Anja Stelzl, Hannes Rolbieski, Mitul Desai, Sarah Bricault, Tobias P. Wörner, Joost Snijder, Arie Geerlof, Helmut Fuchs, Martin Hrabé de Angelis, Albert J. R. Heck, Alan Jasanoff, Vasilis Ntziachristos, Jürgen Plitzko, Gil G. Westmeyer

Supplementary Information

Table of Contents **Page**

1. **Sup. Fig. 1**: Single particle cryo-EM of purified encapsulins and *in vivo* expression. **1**

2. **Sup. Fig. 2**: Further characterization of the size and mass of encapsulins heterologously expressed in HEK293T cells. **2**

3. **Sup. Fig. 3:** Characterization of the cargo loading and the encapsulin subunits purified from HEK293T cells. **3**

4 **Sup. Fig. 4:** Compartmentalization of enzymatic reactions and comparison of the electrophoretic mobility of cargo-loaded encapsulins on BN-PAGE **4**

5. **Sup. Fig. 5**: Expression and iron-loading efficiency of encapsulin variants and modified ferritin-like cargo **5**

6. **Sup. Fig. 6:** Representative tomogram and renderings of encapsulins expressed in HEK293T cells. **6**

7. **Sup. Table 1:** Expression constructs encoding variants of the encapsulin shell as well as various cargo proteins. **7**

8. Supplementary Methods **9**

9. Supplementary References **10**

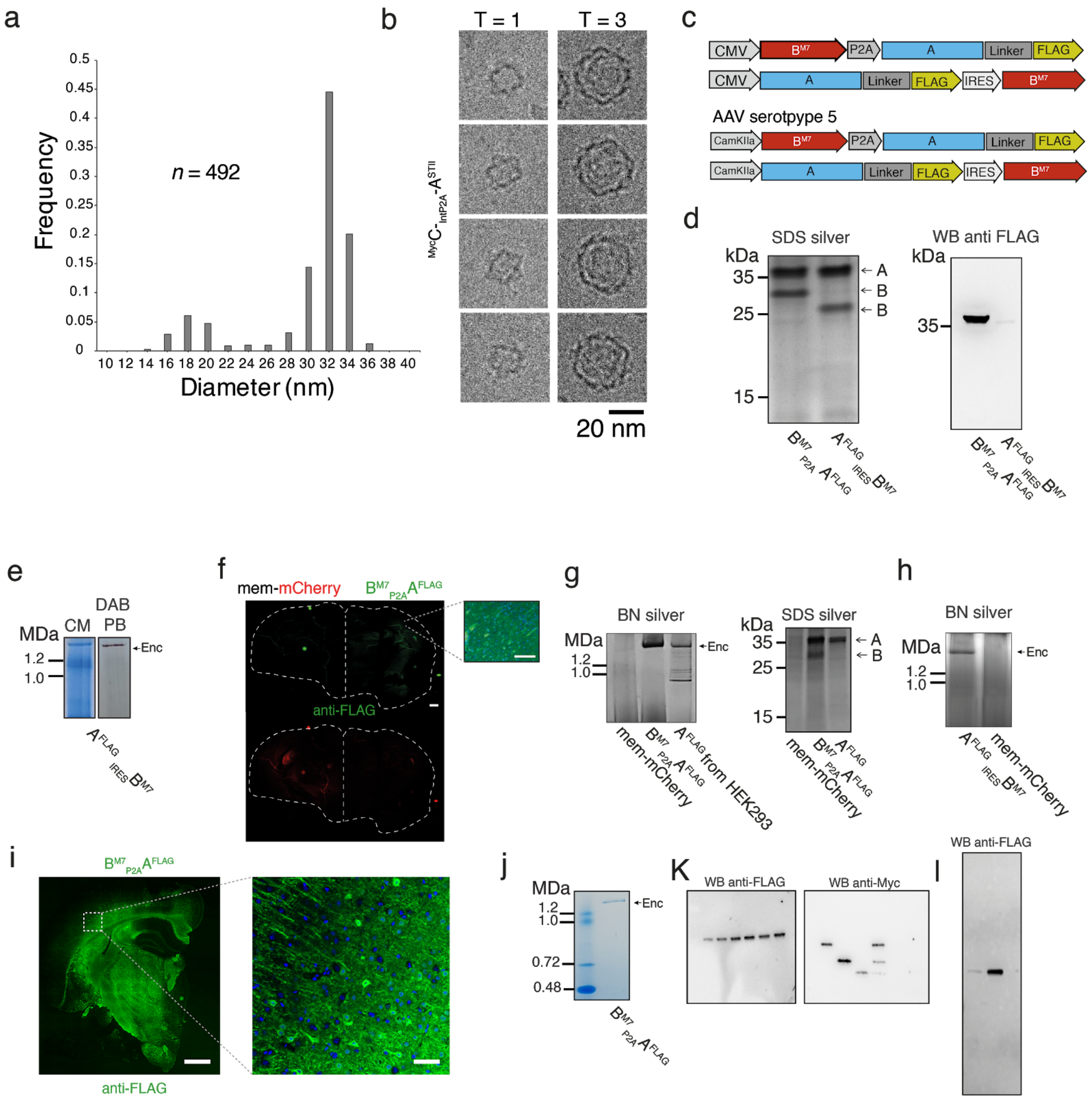

Supplementary Figure 1: Single particle cryoEM of purified encapsulins and *in vivo* expression. **a** Distribution of the diameters measured from single particle cryo-EM images of encapsulins loaded with the native cargo ^{Myc}C ($^{Myc}C\text{-}_{IntP2A}\text{-}A^{STII}$) and purified from HEK293T cells. **b** Corresponding cryo-EM images showing representative encapsulins of the less abundant species with an average diameter of ~18 nm (seen in the histogram in a) and consistent with an assembly of 60 subunits (T=1), as well as the dominant species consistent with the T=3 configuration composed of 180 subunits and an average diameter of 32 nm. **c** Schematic of multi-gene expression construct for co-expression of B^{M7} and A^{FLAG} via a P2A system ($B^{M7}{}_{P2A}A^{FLAG}$) or an internal ribosomal entry site (IRES) site ($A^{FLAG}{}_{IRES}B^{M7}$) in **d** HEK293T cells via transfection (left panel, as shown via a Co-Immunoprecipitation (Co-IP) on a silver-stained SDS-PAGE) and in CHO cells using Adeno-associated virus (AAV) serotype 5 (right panel, as shown on an anti-FLAG western blot (WB)). **e** Coomassie and DAB-enhanced Prussian Blue (DAB PB) stained Blue Native PAGE (BN-PAGE) of whole cell lysates of HEK293T expressing $A^{FLAG}{}_{IRES}B^{M7}$ demonstrating assembly and iron loading. **f** Immunohistochemistry of coronal slices through a mouse brain 3 weeks after localized transduction with an AAV5 encoding $B^{M7}{}_{P2A}A^{FLAG}$ in one hemisphere and membrane-bound mCherry (red) on the contralateral side. Scale bar is 500 µm. The magnified view on the right side shows the presence of the FLAG epitope (green) in neurons. Scale bar is 20 µm. **g** Silver-stained BN-PAGE (left panel) loaded with material from a lysed mouse brain transduced with AAV5 $B^{M7}{}_{P2A}A^{FLAG}$ showing the presence of assembled encapsulin in the hemisphere transduced with $B^{M7}{}_{P2A}A^{FLAG}$. As an additional control, A^{FLAG} encapsulin overexpressed from HEK293T cells was loaded into the right lane. Silver-stained SDS-PAGE of the immunoprecipitated material showed A^{FLAG} around 35 kDa and the cargo molecule B^{M7} below 25 kDa (right panel). **h** Material prepared analogously to that analyzed in panel e and loaded on a BN-PAGE (silver-stained) revealed expression of A^{FLAG} and B^{M7} via an IRES site (instead of the P2A sequence) in murine brain. **i** Immunohistochemistry of coronal slices through one hemisphere of a mouse brain 6 weeks after transduction with an AAV encoding $B^{M7}{}_{P2A}A^{FLAG}$. Left panel: Montaged epifluorescence microscopy images showing the FLAG-epitope on the encapsulins in green. Scale bar is 1 mm. Right panel: Confocal microscopy images of the region identified by the white dashed box showing individual neurons. Nuclear stain (DAPI) is shown in blue. Scale bar is 50 µm. **j** Coomassie-stained BN-PAGE loaded with material from anti-FLAG pull-down from lysates of the other hemisphere of the brain analyzed in panel i. **k** Uncropped raw chemiluminescent imaging data from WB against FLAG and Myc as shown in Fig. 1c. **l** Uncropped raw chemiluminescent imaging data from WB against FLAG as shown in Fig. 6b. The left lane is not shown in the main figure as it was loaded with material from a different clone with lower expression levels.

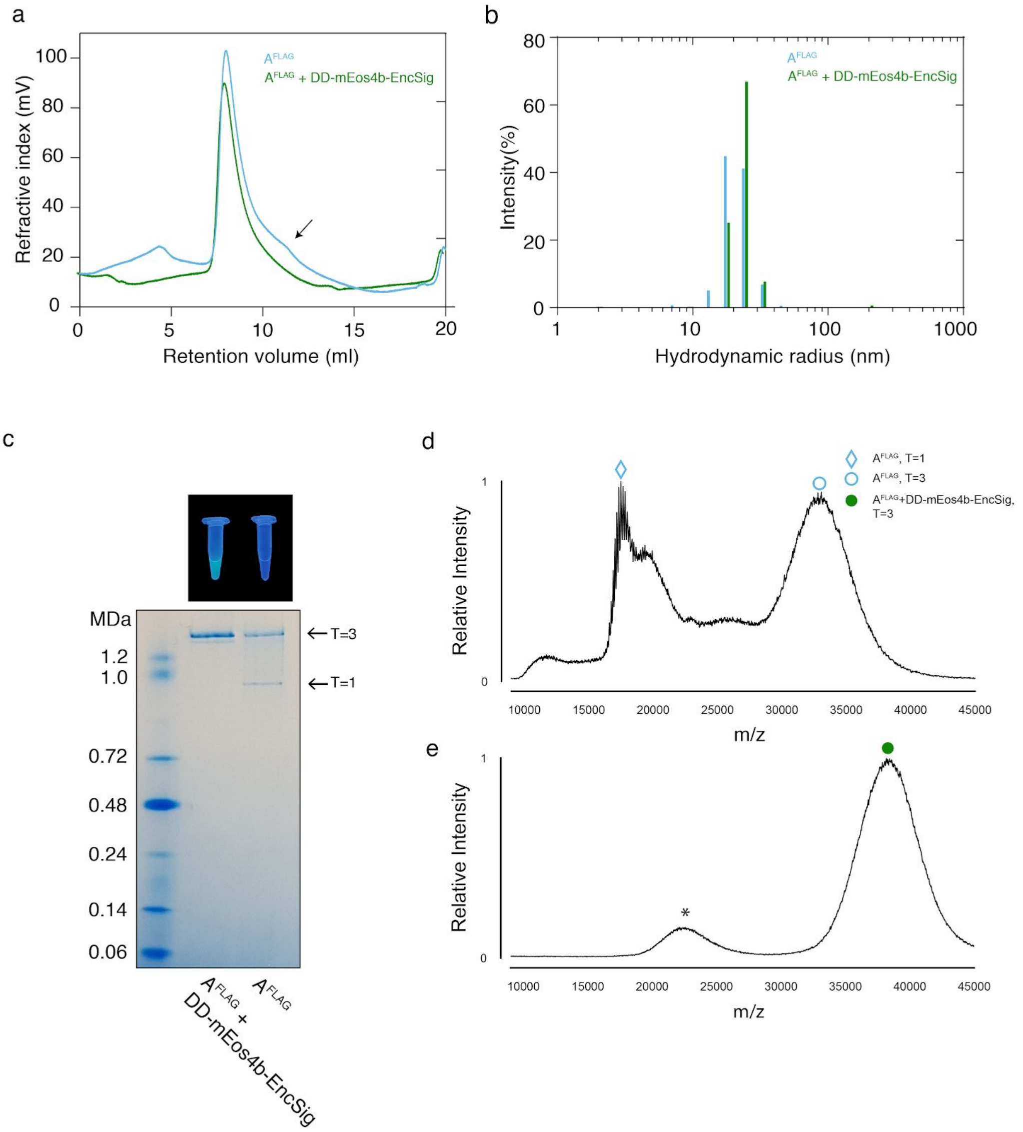

Supplementary Figure 2: Further characterization of the size and mass of encapsulins heterologously expressed in HEK293T cells. We expressed either A^{FLAG} alone or A^{FLAG} together with the fluorescent protein mEos4b (with an FKBP12-derived degradation signal (DD) added) in a 1:1 ratio with the number of shell proteins (DD-mEos4b-$EncSig_{P2A}A^{FLAG}$) in HEK293 cells. We subsequently affinity-purified the assembled encapsulins via agarose affinity beads coupled to anti-FLAG M2 antibodies. **a** We ran both samples described above on size exclusion chromatography (SEC) using a Superose® 6 10/300 GL (from GE) with a separation range from 5 - 5000 kDa connected to an Äkta FPLC. As seen in the refractive index (RI) signals, both samples eluted very close to the void fraction of the SEC column (approx. 8 ml) indicating a Mr of 5 MDa or higher. A more accurate determination of the molecular mass was thus not possible, also because the high molecular weight samples could only be run at relatively low protein concentrations resulting in a noisy baseline in the RI signals and inaccurate Mr values. The RI signal of A^{FLAG} without cargo showed, besides the main peak, an additional peak (black arrow) at approx. 11.5 ml indicating the presence of a less abundant, smaller species. **b** Dynamic light scattering was performed on the same samples to determine the average hydrodynamic radius resulting in 22.7 ± 0.35 nm for A+DD-mEos4b-EncSig (mean and SD over several dilutions from each sample) and 17.1 ± 0.11 nm for A^{FLAG} expressed without cargo, which is consistent with the presence of a smaller species. Samples obtained from an independent expression and purification confirmed an average hydrodynamic radius of 21.1 ± 0.63 nm for A^{FLAG}+DD-mEos4b-EncSig and 19 ± 1.15 nm for A^{FLAG}. The histogram shows the average intensity distribution for the second measurement. **c** BN-PAGE of the purified material specified above. Consistent with the results from SEC, encapsulins loaded with DD-mEos4b-EncSig migrated as a single molecular species above >1.2 MDa, whereas the A^{FLAG} samples showed also a less abundant species running below 1 MDa. The picture (on top) displays reaction tubes containing the samples visualized on an UV-imager to show the fluorescence originating from the mEos4b-filled encapsulins. **d,e** Native mass spectra of the same samples as analyzed by BN-PAGE. **d** The charge state resolved spectrum of the not cargo-loaded encapsulin particles in T=1 symmetry is highlighted at *m/z* 17497 (blue diamond) from which a mass of 1976±0.7 kDa was calculated. The center of the unresolved charge state distribution of the empty encapsulin with T=3 symmetry is labeled with a blue open circle (*m/z* 32918). **e** The center of the unresolved charge state distribution of the T=3 encapsulin loaded with mEos4b is shifted to *m/z* 38291 (green solid circle). The asterisk denotes a known artifact generated by the instrument [1].

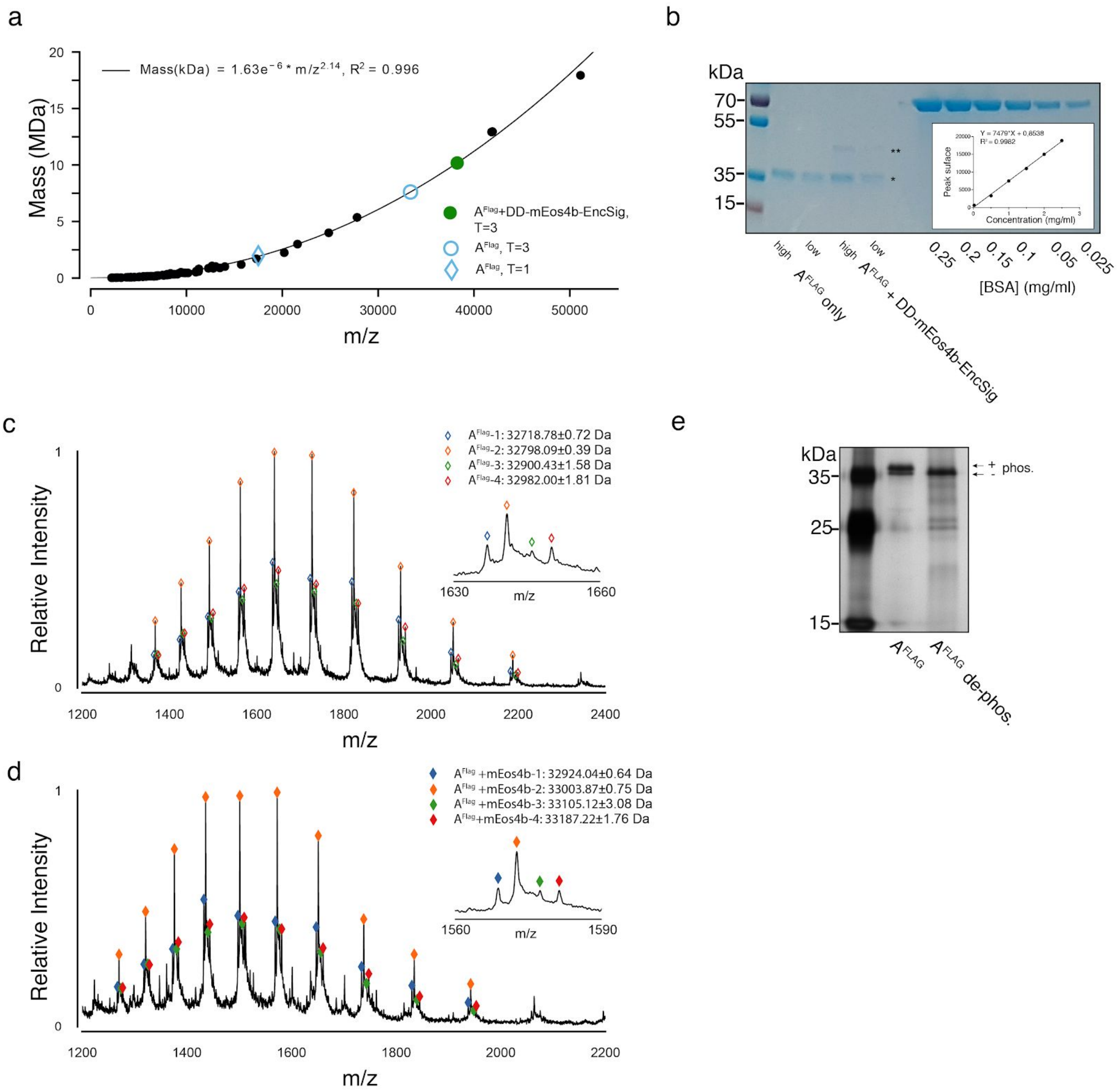

Supplementary Figure 3: Characterization of the cargo loading and the encapsulin subunits purified from HEK293T cells. **a** The black filled circles and black fitted curve shows the relationship between mass and *m/z* that were previously obtained from 77 protein assemblies [2,3]. Based on this relationship and the average *m/z* position (from three technical replicates) that we obtained for the empty (*m/z* 33,379±518) and cargo-filled (*m/z* 38,249±395) encapsulins in T=3 symmetry, we estimated an average mass of 7,611±253 kDa and 10,184±225 kDa respectively from which an average cargo load of 62±8 mEos4b proteins per encapsulin can be calculated. **b** SDS-gel loaded with the purified samples specified above and different concentrations of bovine serum albumin (BSA) as reference protein (* corresponds to the band of A^{FLAG} and ** to band of DD-mEos4b-EncSig with a calculated weight of 41.4 kDa). Intensities of the gel bands were quantified using ImageJ based on the calibration curve (inset) obtained from dilutions of bovine serum albumin (BSA). By relating the calculated protein amounts of A^{FLAG} and DD-mEos4b-EncSig, we calculated a number of 63 cargo proteins per 180-mer encapsulin shell. **c,d** Native mass spectra of A^{FLAG} monomers ejected from the assembled encapsulins upon activation are shown for purified encapsulin shells without cargo proteins (c) and with cargo DD-mEos4b-EncSig (d). For both samples, we can observe several proteoforms which show subsequent mass shifts of around +80 Da, +102 Da and +82 Da. This heterogeneity in mass could be the reason why we were not able to resolve charge states for the encapsulins with T=3 symmetry. The relative mass shifts of ~80 Da were suggestive of phosphorylation which we could experimentally confirm by treatment of purified A^{FLAG} encapsulins with calf intestinal phosphatase (CIP) as shown in panel **e**. The higher molecular weight band on silver-stained SDS-PAGE disappeared after phosphatase treatment, and only the lower molecular weight species remained. When we instead estimated the mass of the T=3 encapsulin from the average mass of an ejected monomer, we obtain a lower mass of 5,947 kDa which would then also result in a higher estimate of the average cargo loading. However, it has been shown that proteins from the host cell can also be unspecifically trapped in the encapsulin lumen without binding via the specific encapsulation tag to the inner surface [4]. This unspecific entrapment could account for the difference in these estimated masses, as could incomplete desolvation in the gas-phase or potentially faulty encapsulin assembly with a subunit number higher than 180.

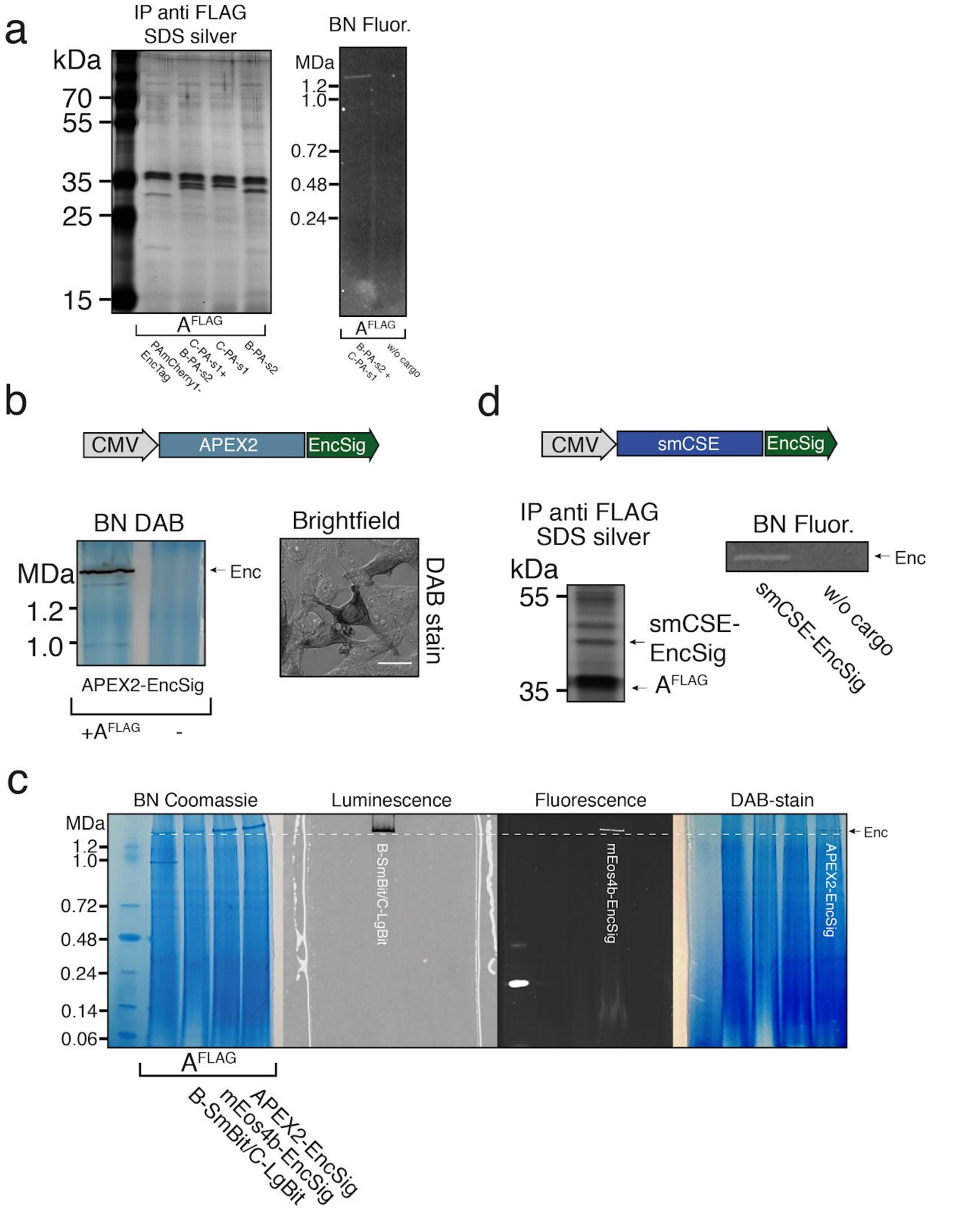

Supplementary Figure 4: Compartmentalization of enzymatic reactions and comparison of the electrophoretic mobility of cargo-loaded encapsulins on BN-PAGE. **a** Non-cropped versions of the gel electrophoresis data shown in Figure 4b. **b** DAB-stained BN-PAGE of whole cell lysates of HEK293T cells expressing APEX2-EncSig with or without A^{FLAG} demonstrating peroxidase activity of APEX2 inside the encapsulin compartment. The brightfield microscopy image (lower panel) shows stained HEK293T cells co-expressing A^{FLAG} and APEX2-EncSig incubated with 0.7 mg/ml DAB and H_2O_2 in 60 mM Tris buffer for 2 min. Scale bar:20 µm. **c** BN-PAGE of whole cell lysates from HEK293T cells expressing A^{FLAG} without cargo or with the engineered cargo proteins B-SmBit/C-LgBit (split luciferase parts fused to B and C), mEos4b-EncSig, APEX2-EncSig. The gel was loaded twice in the same order and cut after the run to yield two identical pieces with the same migration of the proteins into the gel. One half was stained with Coomassie revealing that all encapsulin samples migrated identically irrespective of which cargo was loaded. To confirm the identity of the different cargo proteins, several detection methods were used. First, the other half of the gel was imaged on an UV-imager showing the fluorescent nanocompartment loaded with mEos4b-EncSig. In a second step, this half of the gel was soaked in luciferase substrate solution showing a luminescent band corresponding to the complemented split luciferase inside the encapsulin. Lastly, the gel was washed and incubated in DAB staining solution revealing a dark band corresponding to the lane containing APEX2-EncSig and indicating a DAB polymerization by encapsulated peroxidase activity. The fact that the cargo proteins did not influence the electrophoretic mobility on BN-PAGE indicates that they were encapsulated and did not alter the surface properties (such as shape, hydrodynamic size, and charge) of the nanoshells. **d** Co-IP of the putative cystathionine γ-lyase (SmCSE-EncSig) with encapsulin analyzed by silver-stained SDS-PAGE. The right panel shows a BN gel of encapsulated SmCSE-EncSig with on-gel formation of CdS nanodots after supplementation of 0.5 mM cadmium acetate and 4 mM L-cysteine for 2 h visualized by UV-fluorescence.

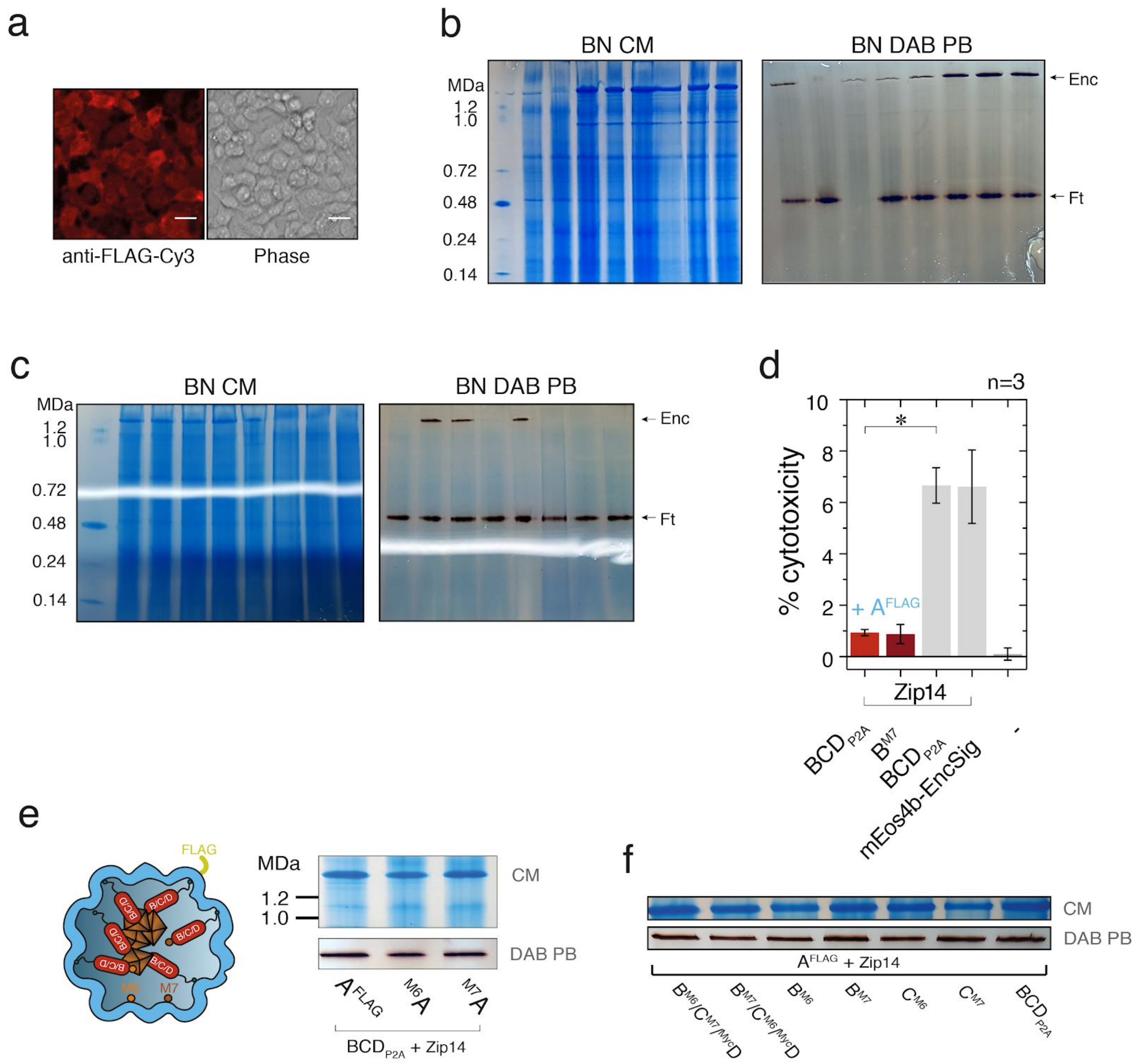

Supplementary Figure 5: Expression and iron-loading of encapsulin variants and modified ferritin-like cargo. **a** Immunohistochemistry against the FLAG epitope showing signal from A^{FLAG} throughout the cytosol when co-expressed with all native cargo proteins B,C, and D in a stable clone (used to generate the data shown in Figure 5 and 6). **b,c** Non-cropped photographs of the Coomassie (CM) and DAB-enhanced Prussian Blue (DAB PB)-stained BN gels shown in Figure 6 c (b corresponds to the upper panel and c to the lower panel). When excess iron was supplied as in this case, we also observed iron-loading of endogenous Ferritin (Ft) at ~480 kDa. Bright horizontal lines across the gels are reflection artefacts during photography. Lanes 1 and 2 in b were loaded with lysates from cells expressing a co-expression construct and un-transfected cells respectively and are unrelated to the focus of Figure 6c. **d** Cytotoxicity assay based on LDH leakage from cells expressing encapsulin with all native cargo (A^{FLAG} + BCD_{P2A}) or just ferritin-like B (A^{FLAG} + B^{M7}) compared to cells without encapsulin expression. Iron-loading of cells was induced by co-expression of Zip14 and supplementation with 2.5 mM FAS for 24 hours. Non-transfected cells (-) served as negative control. The bars represent the mean ± SEM (p=0.0238, Mann Whitney test, n=3 biological replicates). **e** Coomassie (CM) and DAB PB stained BN gel of whole cell lysates of HEK293T cells co-expressing different variants of the nanosphere containing N-terminal tags of peptides reported to aid in iron precipitation (Mms) and BCD_{P2A}. **f** Same analysis conducted on different combinations of native cargo molecules B,C and D modified with C-terminally attached Mms6 (^{M6}) or 7 (^{M7}) peptides. Zip14 was co-expressed and 2.5 mM FAS was supplemented for 48 h for both (a,b).

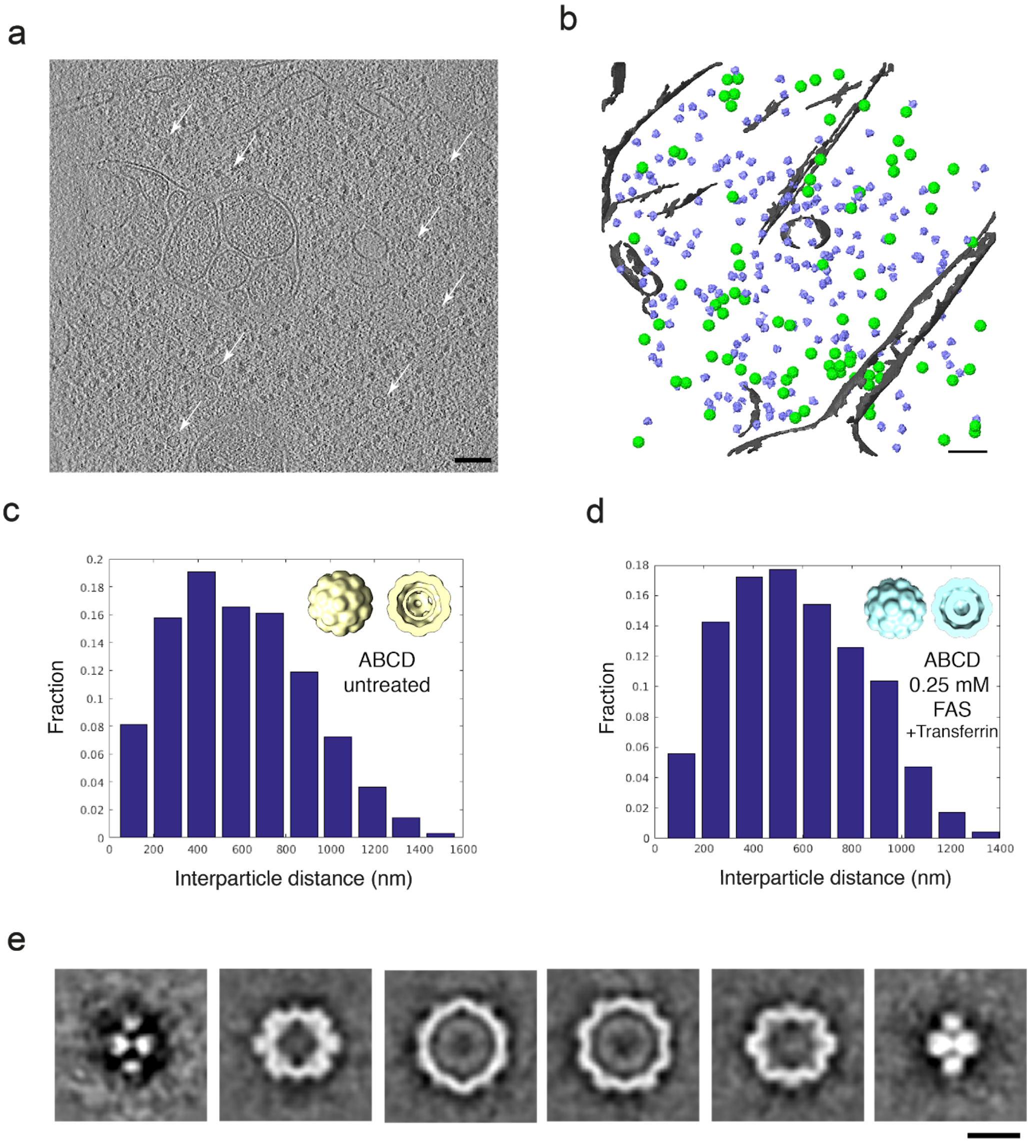

Supplementary Figure 6: Representative tomogram and renderings of encapsulins expressed in HEK293T cells. (**a**) Slice through a cryo-EM tomogram from encapsulins expressed without iron supplementation via the dual promoter construct A^{FLAG};BCD_{P2A}. Scale bar is 100 nm. (**b**) The rendering depicts encapsulins in green and ribosomes in blue. Scale bar is 100 nm. (**c,d**) Histograms showing the mean distance between encapsulins expressed in HEK293T cells without iron supplementation (left, mean distance 589 ± 5 nm) and after addition of 0.25 mM FAS + transferrin (1 mg/ml) iron supplementation for 48 hours (right, mean distance 572 ± 8.1 nm). (**e**) Slices through the averaged encapsulin shell (no symmetry applied) obtained by subtomogram averaging in HEK293T cells expressing the native cargo proteins (ABCD). The average diameter was measured as 31.1 ± 0.1 nm (mean ± SEM, n = 298) from individual 2x binned subtomograms (pixel size 1.368 nm). Scale bar is 20 nm.

Supplementary Table 1: Expression constructs encoding variants of the encapsulin shell as well as various cargo proteins.

**Encapsulin shell proteins**		
pcDNA 3.1 (+) Zeocin MxEncA^{FLAG}	MxEncA-GSG-DYKDDDDK*	EncA: UniProt:MXAN_3556
pcDNA 3.1 (+) Zeocin MxEncA^{StrepTagII}	MxEncA-GSG-WSHPQFEK*	EncA: UniProt:MXAN_3556
pcDNA 3.1 (+) Zeocin BM40-MxEncA^{StrepTagII}	MRAWIFFLLCLAGRALAA-MxEncA-GSG-WSHPQFEK*	EncA: UniProt:MXAN_3556
pcDNA 3.1 (+) Zeocin Mms6ct- MxEncA^{FLAG}	MYAYMKSRDIESAQSDEEVELRDALA-MxEncA-GSG-DYKDDDDK*	EncA: UniProt:MXAN_3556
pcDNA 3.1 (+) Zeocin Mms7ct- MxEncA^{FLAG}	MYVWARRRHGTPDLSDDALLAAAGEE-MxEncA-GSG-DYKDDDDK*	EncA: UniProt:MXAN_3556
**Native cargo proteins**		
pcDNA 3.1 (+) Zeocin MxEncBCD$_{P2A}$	MxEncB-GSG-ATNFSLLKQAGDVEENPGP-MxEncC-GSG-ATNFSLLKQAGDVEENPGP-MxEncD*	UniProt: EncB: MXAN_3557, EncC: MXAN_4464, EncD: MXAN_2410
pcDNA 3.1 (+) Zeocin ^{Myc}MxEncB	MEQKLISEEDL-MxEncB*	EncB: MXAN_3557
pcDNA 3.1 (+) Zeocin ^{Myc}MxEncC	MEQKLISEEDL-MxEncC*	EncC: MXAN_4464
pcDNA 3.1 (+) Zeocin ^{Myc}MxEncD	MEQKLISEEDL-MxEncD*	EncD: MXAN_2410
**Engineered cargo proteins**		
pcDNA 3.1 (+) Zeocin MxEncB-Mms6ct	MxEncB-GGGGSGGGGS-YAYMKSRDIESAQSDEEVELRDALA*	
pcDNA 3.1 (+) Zeocin MxEncB-Mms7ct	MxEncB-GGGGSGGGGS-YVWARRRHGTPDLSDDALLAAAGEE*	
pcDNA 3.1 (+) Zeocin MxEncC-Mms6ct	MxEncC-GGGGSGGGGS-YAYMKSRDIESAQSDEEVELRDALA*	
pcDNA 3.1 (+) Zeocin MxEncC-Mms7ct	MxEncC-GGGGSGGGGS-YVWARRRHGTPDLSDDALLAAAGEE*	
pcDNA 3.1 (+) Zeocin PAmCherry1-EncSig	PAmCherry1-GGGGSGGGGS-LTVGSLRR*	
pcDNA 3.1 (+) Zeocin mEos4b-EncSig	mEos4b-GGGGSGGGGS-LTVGSLRR*	
pcDNA 3.1 (+) Zeocin DD-mEos4b-EncSig	MGVQVETISPGDGRTFPKRGQTCVVHYTGMLEDGKKVDSSRDRNKPFKFMLGKQEVIRGWEEGVAQMSVGQRAKLTISPDYAYGATGHPGIIPPHATLVFDVELLKPE-mEos4b-GGGGSGGGGS-LTVGSLRR*	
pcDNA 3.1 (+) Zeocin MxEncC-PAmCherry1-1-159	MxEncC-GGGGSGGGGS-PAmCherry1-1-159*	
pcDNA 3.1 (+) Zeocin MxEncB-PAmCherry1-160-239	MxEncB-PAmCherry1-160-239*	
pcDNA 3.1 (+) Zeocin MxEncC-LgBit	MxEncC-GGGGSGGGGS-LgBit*	
pcDNA 3.1 (+) Zeocin	MxEncB-GGGGSGGGGS-SmBit*	

MxEncB-SmBit		
pcDNA 3.1 (+) Zeocin APEX2-EncSig	APEX2-LQLPPLERLTLD-GGGGSGGGGS-LTVGSLRR*	
pcDNA 3.1 (+) Zeocin smCSE-EncSig	smCSE- LTVGSLRR*	
pcDNA 3.1 (+) Zeocin ^{Myc}MxEncD-BmTyr	MEQKLISEED-MxEncD-GGGGSGGGGS-BmTyr*	BmTyr: UniProt: B2ZB02

**Other Constructs**		
pCMV-EFYP	EYFP	
pcDNA 3.1 (+) Zeocin mEos4b	mEos4b	
pcDNA 3.1 (+) Zeocin MmZip14^{FLAG}	MmZip14-GGGGSGGGGS-DYKDDDDK*	MmZip14: UniProt: Q75N73
pIRES2-MmZip14^{FLAG}-ZsGreen1	MmZip14-GGGGSGGGGS-DYKDDDDK*	MmZip14: UniProt: Q75N73
pCMV-hHHF	HHF	HHF: UniProt: P02794
**Multi-gene expression/ viral vectors**		
pcDNA 3.1 (+) Zeocin ^{Myc}MxEncC$_{IntP2A}$MxEncA^{STII}	MEQKLISEED-MxEncC-SspDnaE(N159A)-CSCGSGSRGGSG-MxEncA-GSG-WSHPQFEK*	
pcDNA 3.1 (+) Zeocin DD-mEos4b-EncSig$_{P2A}$A^{FLAG}	MGVQVETISPGDGRTFPKRGQTCVVHYTGMLEDGKKVDSSRDRNKPFKFMLGKQEVIRGWEEGVAQMSVGQRAKLTISPDYAYGATGHPGIIPPHATLVFDVELLKPE-GSG-mEos4b-GGGGSGGGGS-LTVGSLRR-GSG-ATNFSLLKQAGDVEENPGP-MxEncA-GSG-DYKDDDDK*	
pBudCE4.1 MxEncA^{FLAG}/BCD$_{P2A}$	CMV:: MxEncA^{FLAG}*, EF1alpha:: BCD$_{P2A}$*	
pcDNA 3.1 (+) Zeocin MxEncB-Mms7ct$_{P2A}$MxEncA^{FLAG}	MxEncB-Mms7ct-GSG-ATNFSLLKQAGDVEENPGP-MxEncA^{FLAG}*	
pcDNA 3.1 (+) Zeocin MxEncA-FLAG$_{IRES}$MxEncB-Mms7ct	MxEncA-FLAG-ECMV-IRES-MxEncB-Mms7ct*	
pAAV-CaMKIIa-MxEncB-Mms7ct$_{P2A}$MxEncA^{FLAG}	MxEncB-Mms7ct-GSG-ATNFSLLKQAGDVEENPGP-MxEncA-FLAG*	
pAAV-CaMKIIa-MxEncA^{FLAG}$_{IRES}$MxEncB-Mms7ct	MxEncA-FLAG-ECMV-IRES-MxEncB-Mms7ct*	

Supplementary Methods

TEM - sample preparation. For single particle cryoEM analysis, we co-expressed StrepTagII-tagged encapsulin shell with the Myc-tagged cargo protein C using the co-expression construct MycC-$_{IntP2A}$-A^{STII}. The StrepTagII-Streptactin system was then used for convenient purification of encapsulins from mammalian cells at a medium scale. Briefly, ~10^8 transfected HEK293T cells were washed with PBS and scraped off cell culture flasks 72 h post-transfection and thoroughly resuspended in 10 ml Buffer W (150 mM NaCl, 100 mM Tris-Cl, pH 8.0) containing protease inhibitor cocktail. Afterwards, the cells were lysed by freeze-thaw-cycling between liquid nitrogen and water at room temperature (4 cycles). Cell debris was spun down for 15 min at 10,000 x g at 4°C. The cleared supernatant was applied to a 1 ml Gravity flow Strep-Tactin®XT Superflow® column (IBA Lifesciences). The column was washed with 5 column volumes and protein was eluted with 1.6 column volumes of Buffer BXT (150 mM NaCl, 100 mM Tris-Cl, pH 8.0, 50 mM Biotin). Before further processing, the purified protein solution was filtered through a 0.45 µm pore filter. Purified protein samples were applied on glow-discharged R1.2/1.3 grids (Quantifoil), plunge-frozen using a Vitrobot Mark IV (FEI, settings: blotforce = 20, blottime = 5 s, temperature = 5 ºC, humidity = 90%) in liquid ethane-propane and stored under liquid nitrogen (LN_2) until use.

TEM - single particle imaging. Single particle grids were imaged at a nominal defocus range of -3 to -1 µm and an EFTEM magnification of 42000x (pixel size 3.42 Å) on a transmission electron microscope (FEI, model: Titan Krios, FEG 300 kV) with a post-column energy-filter (Gatan, model: 968 Quantum K2).
Images were recorded with a direct detection camera (Gatan, model: K2 Summit) using the SerialEM software package[5] using dose-fractionation and a total dose of 40 $e^-/Å^2$ per image. Individual frames were aligned using MotionCor2[6]. Representative areas are shown without further processing.

Cryo-EM - sample preparation. HEK293T cells stably expressing MxEncABCD were grown in 'full growth media' comprising DMEM (Gibco) supplemented with 10% FBS (Gibco), 1% PenStrep (Gibco) and 150 µg/ml Zeocin (Invitrogen) at 37 ºC and 5% CO_2. At 80% confluency, cells were washed with Mg^{2+}-free DPBS (Sigma-Aldrich), dissociated with TrypLE Express (Gibco) and resuspended in full growth media. 150 µL of a suspension containing 150 cells/µL was plated on glow-discharged R1/4 gold grids (200 mesh, Quantifoil) in 4-well dishes and cells were allowed to attach. After 2 h, 2 mL of full growth media were added to the dishes. For iron-loading studies, the media was supplemented with either 0.25 mM FAS and 1 mg/mL transferrin, or 5 mM FAS respectively 12 h after seeding. Cells were plunge frozen on a Vitrobot Mark IV (FEI, settings: blotforce = 10 s, blottime = 10 s, temperature = 37 ºC, humidity = 90%) after an additional 48 h and stored under LN_2 until use. Samples were clipped in modified Autogrids (FEI) and for each grid, thin (100 nm - 180 nm) lamellae were cut from randomly selected cells using a dual beam focused ion beam (FIB) microscope FIB Quanta 3D FEG (FEI) equipped with a Quorum PP3000T cryo-system (Quorum Technologies, Laughton, United Kingdom) and a homemade 360° cryo-stage cooled by an open nitrogen circuit following published protocols[7,8].

Cryo-EM - data acquisition. Cryo tomograms were acquired at a defocus of -5 µm and an EFTEM magnification of 42000x (pixel size 3.42 Å) on a transmission electron microscope (FEI, model: Titan Krios, FEG 300 kV) with a post-column energy-filter (Gatan, model: 968 Quantum K2). Images were recorded with a direct detection camera (Gatan, model: K2 Summit) in dose-fractionation mode and a total dose of ~ 120 $e^-/Å^2$ per tomogram using the SerialEM software package[5]. A dose-symmetric tilt scheme[9] with an angle increment of 2 º between the range of 70 º and -50 º starting at 10 º was used to account for the lamella pre-tilt (~ 10 º). Frames were aligned using motioncorr2[10] and tilt-series alignment as well as tomogram reconstructions were performed in IMOD[11].

Cryo-EM - template Matching and Subtomogram Averaging. *EncA* positions were determined by template matching on 2x binned tomograms (IMOD bin 4, 13.68 Å pixel size) with the PyTom[12] software package. The template was a published structure of EncA purified from E. *coli* without cargo (EMDB-5917) filtered to 50 Å. For ribosome positions, a reference was constructed from ~500 manually picked ribosomes from the data and used for template matching. For each structure, the 400 highest cross-correlation peaks were extracted from the tomographic volumes, and the subtomograms were visually inspected to remove false positives. After initial alignment in PyTom, refinement of subtomogram averages was performed using Relion2.1[13] including normalization and CTF correction with CTFFIND4.1.5[14]. To account for the icosahedral symmetry of the EncA capsules, I3 symmetry was applied in Relion.
Visualization . To enhance the contrast of TEM insets in Fig. 6b, the sum projection of 5 slices around a central slice are shown. EncA capsules were rendered in Chimera[15] (bin 2) and are depicted at the same threshold levels. For visualization of EncA nanoshells in tomographic volumes, membranes were automatically segmented[16] and subtomogram averages placed at positions derived from template-matching that had been confirmed by visual inspection. Cellular volumetric renderings and animations were produced in Amira (Thermo Fisher).

Supplementary References

1. van de Waterbeemd, M. *et al.* Examining the Heterogeneous Genome Content of Multipartite Viruses BMV and CCMV by Native Mass Spectrometry. *J. Am. Soc. Mass Spectrom.* **27,** 1000–1009 (2016).
2. Veesler, D. *et al.* Architecture of a dsDNA viral capsid in complex with its maturation protease. *Structure* **22,** 230–237 (2014).
3. Snijder, J., Rose, R. J., Veesler, D., Johnson, J. E. & Heck, A. J. R. Studying 18 MDa virus assemblies with native mass spectrometry. *Angew. Chem. Int. Ed Engl.* **52,** 4020–4023 (2013).
4. Rurup, W. F., Snijder, J., Koay, M. S. T., Heck, A. J. R. & Cornelissen, J. J. L. M. Self-sorting of foreign proteins in a bacterial nanocompartment. *J. Am. Chem. Soc.* **136,** 3828–3832 (2014).
5. Mastronarde, D. N. Automated electron microscope tomography using robust prediction of specimen movements. *J. Struct. Biol.* **152,** 36–51 (2005).
6. Zheng, S. Q. *et al.* MotionCor2: anisotropic correction of beam-induced motion for improved cryo-electron microscopy. *Nat. Methods* **14,** 331–332 (2017).
7. Rigort, A. *et al.* A 360º Rotatable Cryo-FIB Stage for Micromachining Frozen-Hydrated Specimens for Cryo-Electron Tomography. *Microsc. Microanal.* **16,** 220–221 (2010).
8. Schaffer, M. *et al.* Optimized cryo-focused ion beam sample preparation aimed at in situ structural studies of membrane proteins. *J. Struct. Biol.* **197,** 73–82 (2017).
9. Hagen, W. J. H., Wan, W. & Briggs, J. A. G. Implementation of a cryo-electron tomography tilt-scheme optimized for high resolution subtomogram averaging. *J. Struct. Biol.* **197,** 191–198 (2017).
10. Zheng, S. Q. *et al.* MotionCor2: anisotropic correction of beam-induced motion for improved cryo-electron microscopy. *Nat. Methods* **14,** 331–332 (2017).
11. Kremer, J. R., Mastronarde, D. N. & McIntosh, J. R. Computer visualization of three-dimensional image data using IMOD. *J. Struct. Biol.* **116,** 71–76 (1996).
12. Hrabe, T. *et al.* PyTom: a python-based toolbox for localization of macromolecules in cryo-electron tomograms and subtomogram analysis. *J. Struct. Biol.* **178,** 177–188 (2012).
13. Scheres, S. H. W. RELION: implementation of a Bayesian approach to cryo-EM structure determination. *J. Struct. Biol.* **180,** 519–530 (2012).
14. Rohou, A. & Grigorieff, N. CTFFIND4: Fast and accurate defocus estimation from electron micrographs. *J. Struct. Biol.* **192,** 216–221 (2015).
15. Pettersen, E. F. *et al.* UCSF Chimera--a visualization system for exploratory research and analysis. *J. Comput. Chem.* **25,** 1605–1612 (2004).
16. Martinez-Sanchez, A., Garcia, I., Asano, S., Lucic, V. & Fernandez, J.-J. Robust membrane detection based on tensor voting for electron tomography. *J. Struct. Biol.* **186,** 49–61 (2014).
