# Supplementary material for: Bacterial encapsulins as orthogonal compartments for mammalian cell engineering
Source: Nat Commun. 2018 May 18;9:1990. doi: 10.1038/s41467-018-04227-3 (PMC5959871; doi:10.1038/s41467-018-04227-3)
Supplement: Supplementary file 3 — Description of Additional Supplementary Files [file 41467_2018_4227_MOESM3_ESM.pdf]

Description of Additional Supplementary Files

File Name: Supplementary Movie 1
Description: Fly-through video of reconstructed cryo-ET data from a HEK cell expressing the encapsulin shell (AFlag) as well as the native cargo proteins (BCD). At the end of the movie, encapsulins are highlighted in green and ribosomes in blue. A frame of this video is also shown in Supplementary Fig. 6.
